# Cats with thermal burn injuries from California wildfires show echocardiographic evidence of myocardial thickening and intracardiac thrombi

**DOI:** 10.1038/s41598-020-59497-z

**Published:** 2020-02-14

**Authors:** Ashley N. Sharpe, Catherine T. Gunther-Harrington, Steven E. Epstein, Ronald H. L. Li, Joshua A. Stern

**Affiliations:** 1 University of California, Davis William R. Pritchard Veterinary Medical Teaching Hospital, Davis, CA USA; 2 University of California, Davis School of Veterinary Medicine, Dept. of Medicine and Epidemiology, Davis, CA USA; 3 University of California, Davis School of Veterinary Medicine, Dept. of Surgical and Radiological Sciences, Davis, CA USA

**Keywords:** Cardiovascular biology, Animal physiology, Thromboembolism, Thrombosis, Cardiomyopathies, Heart failure

## Abstract

Recent increases in the prevalence and severity of wildfires in some regions have resulted in an increased frequency of veterinary burn patients. Few studies exist regarding diagnostics and management of burn wounds in veterinary patients and current knowledge is extrapolated from human literature and research models. Post-burn cardiac injury is a common finding and predictor of mortality in human patients and echocardiography is an important tool in monitoring response to therapy and predicting outcome. We describe the notable findings from cats naturally exposed to California wildfires in 2017 and 2018. Domestic cats (n = 51) sustaining burn injuries from the Tubbs (2017) and Camp (2018) wildfires were prospectively enrolled and serial echocardiograms and cardiac troponin I evaluations were performed. Echocardiograms of affected cats revealed a high prevalence of myocardial thickening (18/51) and spontaneous echocardiographic contrast and thrombi formation (16/51). Forty-two cats survived to discharge and 6 died or were euthanized due to a possible cardiac cause. For the first time, we describe cardiovascular and coagulation effects of thermal burn and smoke inhalation in cats. Further studies in veterinary burn victims are warranted and serve as a translational research opportunity for uncovering novel disease mechanisms and therapies.

## Introduction

Thermal burn injury is commonly reported in human populations and occurs in veterinary patients with potentially devastating metabolic, coagulation, respiratory, and cardiovascular consequences^1,2^. In veterinary patients, thermal burns most often occur secondary to accidental or deliberate flame burns (house fires, wildfires, malicious acts), hot water scald injury, automobile engines, external heating devices, or improperly grounded electrocautery^3–6^. Thermal burn injury due to wildfire exposure in veterinary patients is becoming more relevant due to the increased frequency and severity of wildfires in fire-prone areas such as California^7^. The most recent Northern California wildfires were the deadliest in California history and claimed the lives of 86 people and countless animals, while injuring many others^8^.

Significant cardiovascular effects secondary to thermal burn injury and accompanying smoke inhalation have been demonstrated in human populations and experimental animal models^9–12^. Cardiovascular dysfunction in humans secondary to burn injury occurs in two phases starting with an initial resuscitation phase followed by a hyperdynamic, hypermetabolic phase that may persist for up to three years following initial injury^1,9,13–15^. Initially cardiac function is severely depressed due to a reduction in preload from intravascular volume depletion and a multi-factorial reduction in cardiac output. Increases in circulating cytokines, direct cardiomyocyte apoptosis and reduced contractility in *ex-vivo* models are all reported^13,16^. Around 48 hours following the initial injury, a surge in plasma catecholamines results in a hyperdynamic, hypermetabolic response increasing myocardial oxygen demand and perpetuating cardiac stress^9^. These pathologic changes in addition to the marked inflammatory response and abnormal calcium handling secondary to burn injury result in tachycardia, increased cardiac work, systolic dysfunction, and transient myocardial hypertrophy^13,14^.

Smoke inhalation also has profound effects on the respiratory system and severity of inhalation injury is related to the composition of chemical components and particulate matter, as well as the magnitude of exposure and preexisting conditions^17,18^. Many toxic substances are liberated in wildfires including toxic gases like carbon monoxide and cyanide, as well as pulmonary irritants such as acrolein, aldehydes, and many other complex molecules^19^. Additionally, the coagulation cascade is impacted by smoke inhalation and thermal burn injury. Systemic inflammation secondary to the initial thermal injury results in activation of the coagulation cascade and, subsequently, a hypercoagulable state^20,21^. A prospective experimental study of severe burn injury and smoke inhalation injury in sheep suggested triphasic dysregulation of coagulation culminating with hypercoagulability evidenced by platelet activation with increased fibrinogen and depressed anti-thrombin III levels at 96 hours following injury^22^. Direct platelet activation has also been demonstrated as a result of stimulation by pollutants that are present in high concentration in smoke. A study in mice demonstrated that acrolein, an aldehyde present in high concentrations in smoke from wood fire, results in direct platelet activation^23^. These findings demonstrate the widespread effects of smoke inhalation and burn injury on various body systems that should be considered when determining appropriate therapy.

Despite knowledge that significant cardiovascular effects exist secondary to thermal burn injury and smoke inhalation, the current understanding of cardiovascular effects and appropriate therapy in veterinary patients is limited. Anecdotal evidence of clinical findings in veterinary patients suggests that cardiovascular effects including arrhythmias and hypotension may occur in veterinary patients^2^. The majority of clinical effects and treatment of thermal burn injury and smoke inhalation are extrapolated from human populations or experimental animal models^1,6^. Current recommended therapy includes oxygen support, fluid resuscitation, wound management performed under heavy sedation, pain management, and treatment for ophthalmic and neurologic complications secondary to wildfire exposure^1,2,6^.

Cardiovascular function is an important factor when determining appropriate fluid therapy and sedation protocols. Echocardiography is a non-invasive, commonly used tool to assess cardiovascular structure and function in veterinary patients. Echocardiographic assessment of cardiac function is an important tool in guiding therapy and predicting outcome in human burn patients^24–26^ and may prove beneficial in the care and management of cats exposed to wildfires. Younan et. al. found that echocardiographic evidence of systolic dysfunction in human trauma and burn patients was associated with a four-fold increase in in-hospital mortality^24^. A previous study in mice with experimentally-induced severe burn injury, compared *in vivo* echocardiography and *in vitro* physiologic measurements. This study found that echocardiography paralleled *in vitro* results and confirmed burn-related myocardial contractile dysfunction beginning at 12 hours post-burn and persisting for 72 hours^27^. The findings of these studies suggest that cardiovascular dysfunction secondary to burn injury is evident and may have important implications for therapy and prognosis.

The cardiac biomarker, cardiac troponin I (cTnI) has also been evaluated as a marker of severity of post-burn cardiac injury in human patients^28,29^. Cardiac troponin I is a unique cardiac biomarker originating from the cardiac contractile unit that is conserved across many species and released into circulation after direct cardiomyocyte damage. Cardiac troponin I has detectable peripheral concentration at 2–3 hours post-injury and a peak concentration at 18–24 hours following cardiomyocyte damage^30^. Human burn patients with no pre-existing cardiac conditions had fluctuating elevations in cTnI following burn injury with elevations as early as 10 hours and peak cTnI concentrations at day 7–13 following injury. Normalization of cTnI occurred after excision of eschar formation and administration of cardiovascular support^31^. A separate retrospective evaluation demonstrated an increased risk of death associated with a high cTnI result^28^.

The goal of this study was to prospectively evaluate and report echocardiographic changes that occur in naturally-acquired, thermal burn injury and smoke inhalation in a population of cats presented to a referral institution following exposure to the California wildfires. We hypothesized that clinically evident cardiovascular changes would be appreciated on echocardiogram secondary to burn injury and smoke inhalation in this population.

## Results

A total of 56 cats were presented to the University of California, Davis William R. Pritchard Veterinary Medical Teaching Hospital (UCD-VMTH) for evaluation during the Tubbs fire (n = 23) and Camp fire (n = 33) in 2017 and 2018, respectively for evaluation and treatment of thermal burn injuries. The cats were presented 5–17 days from the start date of the Tubbs wildfire and 4–9 days from the start date of the Camp fire. Some cats affected were treated at outside institutions prior to transferring to the UCD-VMTH. Five cats were excluded from echocardiographic evaluation due to extensive thoracic wounds (n = 1), euthanasia on intake (n = 1) and temperament precluding evaluation without sedation (n = 3), leaving 51 cats for echocardiographic evaluation (32 male, 19 female). Fifty cats were presumed to be domestic shorthair or domestic longhair cats but breed identification was difficult due to burn wounds and unknown history. One Persian cat was presented. Additionally, age was difficult to determine, but all cats included were considered adult. The median weight of the patient population was 4.1 kg (Interquartile range (IQR): 3.43–4.6 kg). Based on subjective assessment of images and medical record notes, 8 cats had mild burns, 14 cats had moderate burns, and 29 cats had severe burns. Cardiac troponin I was performed in the Tubbs fire cohort and demonstrated a median cTnI of 0.07 ng/ml (IQR: 0.02–0.21 ng/ml, Reference range: <0.03–0.16 ng/ml)^32^. Four of the 5 cats with a cTnI > 0.16 ng/ml died or were euthanized. All cats with cTnI > 0.16 ng/ml had significant echocardiographic or necropsy findings including left ventricular (LV) thrombus with relative systolic dysfunction, focal myocardial thickening (MT) and wall thinning (cTnI = 2.11), LV thrombus with MT (cTnI = 1.7), dilated cardiomyopathy (DCM) phenotype with congestive heart failure (CHF) (cTnI = 1.33), high grade second degree atrioventricular block with DCM phenotype and biventricular CHF (cTnI = 0.34), MT with severe left atrial (LA) dilation and DCM phenotype (cTnI = 0.23).

**Figure 1:**
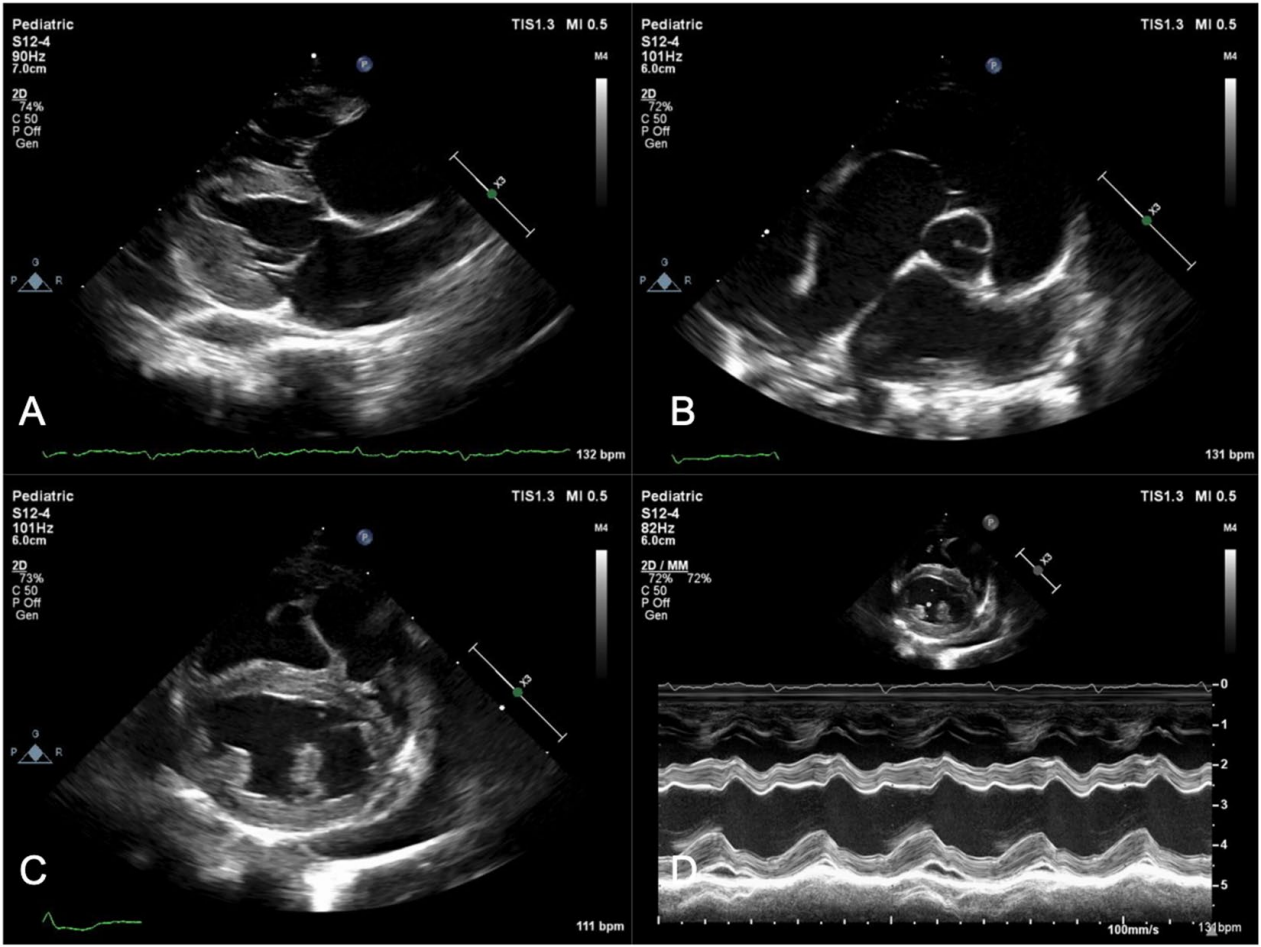
Echocardiographic images from a representative feline patient. (**A**) Demonstrates a right parasternal long-axis 4-chamber view and (**B**), a right parasternal short-axis basilar view both depicting severe biatrial enlargement with scant pericardial effusion. Two-dimensional imaging (**C**) and M-mode (**D**) from the same feline patient at the level of the left ventricular papillary muscles. There is equivocal myocardial thickening, left ventricular dilation, systolic dysfunction (Fractional shortening = 27%), and decreased septal wall motion on M-mode. Necropsy findings in this patient demonstrated dissecting fibrosis with multifocal myonecrosis and hypertrophy.

Echocardiographic findings are summarized in Table 1. The median heart rate at the time of initial cardiac evaluation was 20 $\pm$ 8 (193–224) beats per minute (bpm) (Reference range: 140–240 bpm)^33^. Echocardiographic examination demonstrated MT in 18/51 (35%) of cats on initial evaluation. An additional six cats (12%) had equivocal MT and 2/51 (4%) cats had MT and concurrently decreased left ventricular internal dimension at end diastole (LVIDd) suggestive of pseudohypertrophy. Spontaneous echocardiographic contrast with or without organized thrombus formation (SEC $\pm$ T) was seen in 16/51 cats (29%) on initial presentation (Video 1). Of these 16 cats, 7 had an observed organized thrombus and 9 had no evidence of organized thrombus formation. The left auricular flow velocity was greater than 25 cm/s in all patients (61.6 $\pm$ 18  m/s). A total of 15/51 (29%) cats had left atrial enlargement [left atrium in long axis (LA lax) 1.43 IQR: 1.28–1.59, left atrial to aortic root ratio (LA/Ao) 1.43 IQR: 1.32–1.60]. Additional findings included mitral regurgitation (4/51), tricuspid regurgitation (6/51), hyperechoic papillary muscles or endomyocardium (21/51), and hyperdynamic systolic function (7/51). No cats had systolic dysfunction at initial examination. Fractional shortening was 52.9 $\pm$ 10.3 in exposed patients compared to 58.4 $\pm$ 6.6 in control cats (p = 0.0073). Fourteen cats (27%) were noted to have relative systolic dysfunction. These characteristic echocardiographic findings are depicted in Video 1 and Figure 1.

**Table 1:** Overview of echocardiographic findings in 51 cats with thermal burn injuries and smoke inhalation from California wildfires.

Echocardiographic finding	Initial examination (n = 51)	Follow-up examination (n = 38)
Myocardial thickening	18 (35%)	12 (32%)
Equivocal myocardial thickening	6 (12%)	7 (18%)
Spontaneous echocardiographic contrast	16 (31%)	2 (5%)
Thrombus formation	7 (14%)	3 (8%)
Valvular regurgitation	8 (16%)	4 (11%)
Hyperechoic myocardium	21 (41%)	17 (45%)
Atrial enlargement	15 (29%)	10 (26%)
Congestive heart failure	5 (10%)	2 (5%)
Systolic dysfunction	0 (0%)	2 (5%)
Relative systolic dysfunction	14 (27%)	8 (21%)

Follow-up echocardiographic examination was performed in 38 cats. Follow-up examinations were not performed in all cats due to discharge from the hospital, patient death, or euthanasia. The mean heart rate at follow-up examination was 204 ± 25 bpm. There was MT present in 12/38 cats (32%) and equivocal MT in 7/38 (18%) at follow up. Spontaneous echocardiographic contrast or thrombi were present in 4/38 (10%) with a mean left auricular flow velocity of 64 ± 18 cm/s. Left atrial enlargement was present in 10/38 cats (26%) with a mean LA/Ao of 1.47 $\pm \,0.25$.

**Figure 2:**
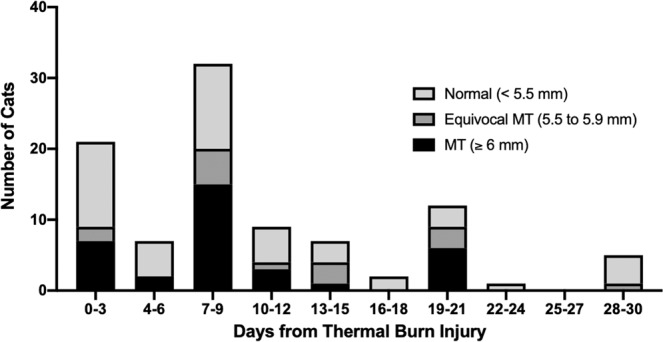
Prevalence of myocardial thickening (MT) and equivocal MT over time. Date of initial thermal burn injury was defined as three days following the official fire start.

**Figure 3:**
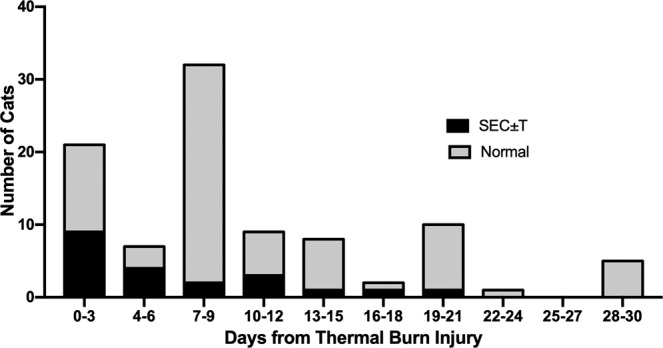
Prevalence of spontaneous echocardiographic contrast +/− thrombus formation (SEC ± T) over time. Date of initial thermal burn injury was defined as three days following the official fire start.

Evaluation of MT over time **(**Figure 2) demonstrated a bimodal distribution with the highest proportion of cats demonstrating MT at day 7–9 (63%) and day 19–21 (75%) from proposed burn date. Spontaneous echocardiographic contrast and presence of intracardiac thrombi demonstrated an increased incidence of SEC $\pm$ T within the first 72 hours from proposed burn date with a significantly reduced incidence at the second evaluation (P = 0.02) (Figure 3). A total of six cats were determined to be in CHF during hospitalization (11.8%). Five cats (10%) were diagnosed with CHF on initial echocardiographic evaluation, and two were determined to be in CHF on follow-up examination (one persistent CHF, one new diagnosis). All patients that developed CHF and all patients that died or were euthanized had severe burns. The only significant association with burn severity identified was patient death (P = 0.0135).

A total of 42 cats (82%) survived to hospital discharge. One patient had a cardiac arrest after administration of ketamine for wound debridement and cardiopulmonary resuscitation was performed with successful return of spontaneous circulation, which prompted further echocardiographic evaluation. This cat was later euthanized. Six cats (12%) died or were euthanized due to cardiac causes. Three cats (6%) died or were euthanized due to non-cardiac causes (severe wounds complicated by diabetes mellitus, acute lung injury, and anuric renal failure). In most cases, election of humane euthanasia or patient death was multifactorial due to reasons including repeated episodes of congestive heart failure (pleural effusion) (n = 2), arrhythmias (high-grade second degree AV block) with progression to peri-arrest (agonal breathing) and perceived pain with abnormal vocalization at which time thromboembolic disease was suspected (n = 1), inappetence and lateral recumbency with associated decompensated CHF (pericardial effusion) (n = 1), decompensated CHF (pericardial effusion) with concurrent renal insufficiency and severe burn wounds (n = 1), uncontrolled diabetes mellitus with severe burn injuries (n = 1), acute tachypnea and sudden death (n = 1), respiratory distress with no echocardiographically apparent cardiac changes consistent with acute lung injury (n = 1), anuric renal failure (n = 1) and progressive anemia with decompensated CHF (pleural effusion, with B-lines consistent with pulmonary edema) (n = 1). The patient that acutely died after developing tachypnea was positive for feline calicivirus with evidence of disseminated intravascular coagulation that likely contributed to decline. Based on necropsy findings, septicemia from severe burn wounds could not be entirely excluded due to similar characteristic findings. Necropsy was not performed for all patients who died or were euthanized, making the cause of death speculative in some cases. Furthermore, diagnostics were limited to only what was deemed necessary to the emergent care of patients, so not all had all diagnostics to support a pre-mortem diagnosis of septicemia or pneumonia (i.e. thoracic radiographs and complete blood counts were not available for most patients).

**Figure 4:**
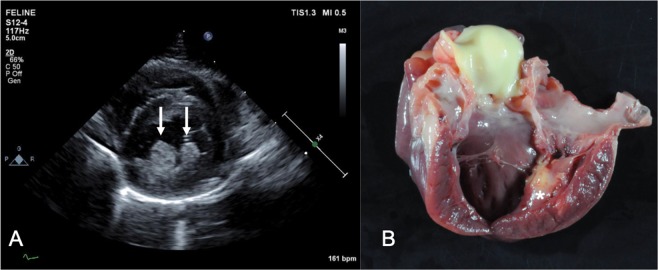
Echocardiographic (**A**) and gross pathology (**B**) images from one feline patient. The echocardiographic image depicts the right parasternal short-axis view at the level of the papillary muscles. The papillary muscles are prominent and hyperechoic (arrows). Gross pathology image demonstrated a pale multi-nodular focus within the papillary muscle (*) and extending through the left ventricular free wall. Histopathology of this region demonstrated severe myocarditis with intralesional thrombi and coccoid bacteria. Photo courtesy of Dr. Melissa Roy.

Post-mortem evaluation was performed in five cats. All gross pathologic examinations demonstrated severe burns. Notable gross cardiovascular findings included: circumscribed areas of myocardial pallor (n = 2), ventricular dilation (n = 1), and effusion (n = 4). One heart had tan to white foci within a LV papillary muscle extending through the adjacent LV free wall. Histopathologic evaluation of this area demonstrated severe, neutrophilic, lymphocytic and histiocytic myocarditis with intralesional thrombi and coccoid bacteria (Figure 4). Additional histopathologic findings included myofiber atrophy (n = 2) myocardial edema (n = 1), interstitial fibrosis demonstrated with Trichrome staining (n = 2), multifocal myonecrosis and hypertrophy (n = 1), and fibrinous pericarditis (n = 2). One heart with gross concentric hypertrophy was histologically unremarkable. Mean heart weight was 20.2 ± 2.5 grams or 0.46 ± 0.10% body weight (Reference range: 0.28–0.88%)^34^. Left ventricular free wall, interventricular septum and right ventricular free wall gross measurements (mm) were 7.2 ± 1.6, 6.6 ± 1.8, and 2.4 ± 0.55 mm, respectively. There was evidence of interstitial pneumonia (n = 2) or bronchopneumonia (n = 1) present at necropsy evaluation in 3/5 patients. The remainder of the necropsy findings are beyond the scope of this manuscript.

**Figure 5:**
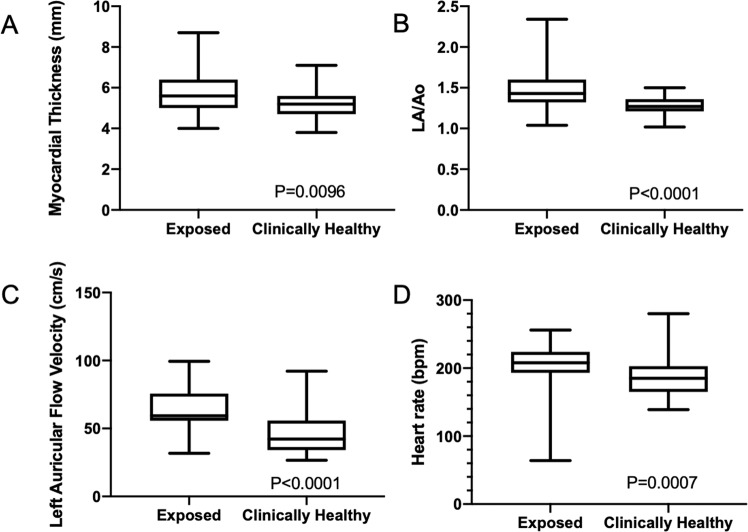
Box and whisker plots depicting comparison of data between clinically healthy population (n = 35) and wildfire-exposed population (n = 51). The box represents the interquartile range with the horizontal line representing the median. The whiskers extend to the minimum and maximum values. The myocardial thickness (**A**) and left atrial to aortic root ratio (LA/Ao) (**B**) were significantly elevated in the wildfire-exposed population. The heart rate (**C**) and left auricular flow velocity (**D**) were also significantly elevated in the affected population, likely consistent with an increased output state following burn injury.

The clinically healthy population consisted of 35 domestic cats with no known history of thermal burn injury. Mean body weight was 5.33 $\pm$ 0.92 kg and the patients were 7.51 $\pm$ 5.0 years old. Heart rate of the exposed cats during initial examination was significantly elevated from control cats who had a median heart rate of 185 (165–203 bpm, P = 0.007). The mean maximum wall thickness from the first evaluation was 5.6 mm (5.0–6.4 mm), which was significantly elevated from the clinically healthy population (5.2 $\pm$ 0.73, P = 0.0096). Left atrial to aortic root ratio was significantly higher in the exposed population (P < .0001). Left auricular flow velocity was also significantly elevated in the exposed group (<0.0001). These findings are demonstrated in Figure 5. The clinically healthy cats had significantly larger body weights than the wildfire exposed cats (P < 0.0001).

Cats affected by thermal burn injury from wildfire exposure were approximately 4.5 times more likely to have MT (95% CI, 1.71–11.7, P = 0.0023) and 3.1 times more likely to have SEC $\pm$ T (95% CI,1.56–6.29, P = 0.0013) relative to clinically healthy cats.

## Discussion

Our study demonstrates the characteristic echocardiographic findings in cats with naturally-occurring thermal burn injury and smoke inhalation from California wildfires. Common echocardiographic findings included MT, SEC $\pm$ T, CHF, and relative systolic dysfunction. Significant systolic dysfunction was not appreciated in our patient population which may be explained by the elapsed time from initial injury to evaluation as the hypodynamic phase of cardiac dysfunction is only present for approximately the initial 48–72 hours in thermal burn injury animal models and human studies^1,35,36^.

Myocardial thickening was present in over half the cats (51%) during at least one evaluation. Potential etiologies of MT include occult cardiomyopathy, myocarditis, myocardial edema or hypertrophy secondary to massive catecholamine surges following injury. Previous studies demonstrated a prevalence of occult hypertrophic cardiomyopathy (HCM) in approximately 15% of apparently healthy cats evaluated by echocardiography^37–39^. This is consistent with the clinically healthy population in this study (11.4%) and suggests that the MT appreciated in the evaluated population was not solely due to occult HCM. A bimodal distribution pattern was present when MT was evaluated over time with a peak at day 7–9 and day 19–21 from proposed burn date. This may represent pathologic variations in ventricular wall thickness over time or may be artifactual due to discrepancies in determining precise time from initial injury despite attempts to standardize this. Additionally, serial echocardiography demonstrated improvement and resolution of MT in some cases evaluated (n = 8) as well as increases in MT in other cases (n = 6) over time, suggesting a dynamic, transient myocardial thickening (TMT). Novo Matos *et al*. previously demonstrated the phenomenon of TMT in feline patients following antecedent stressful events. In that study, TMT with CHF and elevated cTnI values were noted and normalized over time, leading to an excellent prognosis for survival and discontinuation of all cardiac medications^40^. Myocardial edema was the suspected mechanism in TMT reported in that study and has also been documented via magnetic resonance imaging and histopathology in human patients with myocarditis and takotsubo cardiomyopathy^40,41,42^. Takotsubo cardiomyopathy is defined as a transient cardiomyopathy induced by severe emotional or physical stress which has also been reported in multiple human patients after burn injury. It is suspected to occur secondary to the massive surge in plasma catecholamines that accompanies burn injury^43,44^. Typical echocardiographic findings include apical ballooning and transient wall motion abnormalities, however atypical cases have been reported in humans and are characterized by TMT^40,41,45–46^. Furthermore, a previous study in rats with experimentally-induced non-severe burn injury demonstrated systolic dysfunction and MT that was ameliorated with beta-blockade, providing additional support that a component of catecholamine-induced MT is present following burn injury^47^.

Due to suspicion for myocarditis secondary to burn injury and smoke inhalation, cTnI was performed in all cats in the Tubbs cohort and was within normal limits for all but 5 patients. However, all patients with elevated cTnI had significant echocardiographic findings and 80% of patients with elevated cTnI ultimately died or were euthanized. The lack of widespread cTnI elevation is suspected to be a function of the short half-life of cTnI and time of sample collection. Cardiac troponin I has a reported half-life of approximately 2 hours^30^ and animal burn models have demonstrated that peak cTnI levels occur around 24 hours post-burn^31,36^. Given the timeline of patient presentation, active myocarditis and/or myocardial damage may have resolved by the time of evaluation. Persistent MT despite normal cTnI levels can consequently be explained by replacement of inflammatory cells with myocardial edema and/or fibrosis that was appreciated on histopathology. Necropsy findings for the available cats demonstrated histopathologic findings of myocarditis, myocardial fibrosis and myocardial edema which supports the aforementioned etiologies for MT.

Spontaneous echocardiographic contrast and thrombi formation was another common finding in the evaluated population and incidence decreased significantly over the study period. Spontaneous echocardiographic contrast (SEC) is described as dynamic, coordinated swirling of blood visible without the administration of an exogenous contrast agent. It is a common finding and predictor of morbidity and mortality in human patients with left atrial enlargement and is associated with increased incidence of left atrial thrombus formation and thromboembolic events^48^. Spontaneous echocardiographic contrast has also been associated with increased risk of death in cats with cardiomyopathy^49^. The finding of SEC can be the result of increased red blood cell aggregation from interaction with plasma proteins such as fibrinogen with low shear rates and can also occur secondary to platelet-leukocyte activation and aggregation^48,50^. Spontaneous echocardiographic contrast and intracardiac thrombi are likely secondary to the hypercoagulable state following burn injury. Direct platelet activation induced by dioxins, found in particulate matter and generated by combustion of organic compounds, may play a role in the presence of SEC $\pm$ T seen in this patient population^51^. A treatment protocol was developed for the patient population and may have contributed to the clinical improvement witnessed at consecutive evaluations. No known thromboembolic complications occurred in evaluated patients. Further studies are needed to evaluate the coagulation abnormalities present and therapies warranted for veterinary patients affected by thermal burn and smoke inhalation.

During hospitalization, a total of six cats (12%) progressed to CHF as defined by atrial enlargement with evidence of cavitary effusion (ascites, pleural and/or pericardial effusion) or pulmonary edema. These cats demonstrated characteristic clinical signs of tachypnea and respiratory distress prior to therapy. The pathophysiology of CHF in feline burn patients is likely multi-factorial and related to the reported cardiovascular effects of thermal burn injury accompanied by fluid therapy. Myocardial dysfunction following thermal burn injury is characterized by both systolic and diastolic dysfunction. The combination of direct myocardial depressant effects and increased pulmonary artery resistance from smoke inhalation injury resulting in secondary right ventricular dysfunction contributes to the development of biventricular failure in humans^14^. While severe systolic dysfunction was not noted on echocardiogram in the evaluated population, diastolic dysfunction is suspected based on the presence of myocardial thickening. Unfortunately due to tachycardia and resultant E/A fusion, diastolic function was difficult to objectively assess in the given population. Pulmonary capillary hyperpermeability due to smoke inhalation and potential over-resuscitation with fluids may also contribute to pulmonary edema present in this patient population^2^.

A number of inherent limitations exist due to the nature of the study and emergency presentation of patients. The study included two patient cohorts from different fires, one year apart. The exact nature and chemical composition of particulate matter may have differed between fires and the exact burn date and time for each cat is unknown, although attempts at standardization were made. The authors acknowledge that this estimation may be inaccurate given variations in rescue times and the propensity of wounded animals to hide for unknown periods following initial injury. The echocardiograms were also performed by three investigators which may contribute to variations^52,53^. Furthermore, due to the nature of presentation and use of disaster relief funds, diagnostics performed were limited to those directly impacting the emergent care of burn patients so all concurrent systemic diseases (hyperthyroidism, prothrombotic conditions, hypertension) were not excluded in all cats. In addition, the short follow-up period is limiting and a more extensive longitudinal evaluation may provide additional valuable insight into the progression or resolution of cardiovascular findings.

An additional limitation in veterinary medicine is the lack of an approved system for estimation of burn severity in animals to correlate burns to clinical findings. In human patients, the total body surface area (TBSA) affected by the burn is determined by two scales including the “Rule of Nines” and Lund-Browder chart which have not been validated in veterinary patients^1^. Calculation of the TBSA is relevant as previous studies demonstrate that patients with severe burn injury as defined by a TBSA > 20–30% are at risk for burn shock and systemic effects of burn injury, whereas systemic effects are rarely reported in local burn injury^1,2,27^. The majority of the cats presented here had an estimated <20% TBSA. The limitation in accurately identifying TBSA in cats may also have some role in the lack of identifiable associations between burn severity and study variables. Alternatively, type II error due to the small number of patients with mild burn injuries may also explain these findings. Patient death was associated with severe burn injuries in this study, as expected.

Despite the limitations of our study, important findings were demonstrated by observation of echocardiograms performed on cats affected by California wildfires. Severe burn injury and resulting cardiovascular derangements in human patients contribute to the development of sepsis, multiple organ dysfunction and death^14^. Therefore targeting mechanisms of cardiovascular dysfunction may serve to reduce complication rates and improve prognosis in veterinary patients. Future directions include further evaluation and establishment of a comprehensive classification of burn severity and cardiovascular dysfunction in veterinary patients. Additional studies are warranted into the mechanisms of hypercoagulability in these patients as well as appropriate treatment recommendations to prevent potential cardiovascular and thromboembolic complications.

This study provides an outline of the clinically relevant echocardiographic findings in feline thermal burn patients naturally exposed to California wildfires. The underlying mechanism for the cardiovascular findings cannot be definitively proven, however, most findings could be explained by the hemodynamic consequences of burn injury and smoke inhalation, including catecholamine-induced cardiovascular dysfunction exacerbated by concurrent respiratory derangements, coagulation dysfunction and fluid resuscitation attempts. This study highlights the paucity of data available concerning the cardiovascular effects and recommended monitoring in veterinary burn patients and inspires further investigation. Finally, this study highlights the clinical and pathophysiologic similarities between feline smoke inhalation and burn injury patients to humans with similar exposures suggesting a truly translational opportunity for each species to inform future mechanistic and therapeutic research.

## Materials and Methods

### Animals and study design

The study was a prospective observational cohort study. Clinical feline patients were enrolled in this study in accordance with the Institutional Animal Care and Use Guidelines of the University of California Davis (IACUC) (Cats exposed to wildfires were clinical patients treated according to best veterinary practice standards and control cats which underwent cardiovascular screening examinations were enrolled as part of IACUC protocols #20095 and #21037). All experimental protocols were approved by the IACUC of the University of California Davis and all methods were carried out in accordance with relevant guidelines and regulations for care and use of laboratory animals. Cardiac Troponin I samples were only taken from cats in which owner consent was achieved and all other care was considered standard of care in the treatment of the cats burn injuries. All cats presented to the UCD-VMTH for further evaluation and treatment of thermal burn injury and smoke inhalation injury secondary to the Tubbs fires (2017) and Camp fires (2018) were enrolled. Prior environmental, medical history and documentation of thermal burn injury and smoke inhalation was largely unknown. Therefore, an estimated burn date was established as the third day following the official fire start as determined by the California Department of Forestry and Fire Protection incident reports. After presentation, each cat was managed by a veterinarian at the UCD-VMTH. Each cat received standard of care for thermal burn injury, as indicated by the extent, location, and severity of injury. Burn severity was determined by evaluation of physical examination notes and pictures present in the medical record. Superficial burns requiring minimal to no bandaging were considered mild, while burns covering large surface areas or resulting in bone exposure were graded severe. Burns described as moderate or second degree were graded moderate. Treatment often included analgesia, antimicrobial therapy, fluid therapy, nebulization, supportive care, ophthalmic medications and sedation with debridement and bandage changes as warranted. Specific medications received at UCD-VMTH were based on individual patient clinical assessment and primary clinician preference and may have included the following: analgesia (buprenorphine, methadone, hydromorphone, robenacoxib), sedation (dexmedetomidine, ketamine, midazolam, alfaxalone), antimicrobials (amoxicillin-clavulanate, cefovecin, ampicillin-sulbactam, orbifloxacin), topical ophthalmic mediations (ophthalmic lubricant, erythromycin, terramycin, tropicamide, atropine), gastrointestinal support (maropitant, ondansetron, mirtazapine, feeding tubes), cardiac medications (furosemide, pimobendan, calcium gluconate), anti-coagulant medications (clopidogrel, aspirin) and topical wound care (silver sulfadiazine ointment or medical grade honey). Cats with burn injuries of the thorax, and/or a temperament that precluded echocardiogram without sedation, were excluded. Initial echocardiograms were completed within 1–7 days after presentation to the hospital (initial exam) and 7–14 days after the initial exam (follow-up exam). Cardiac evaluation was indicated due to the auscultation of heart murmurs in many of the fire victims on initial physical examination, coupled with the necessity for repeated events of heavy sedation. Due to the high incidence of occult cardiomyopathies in feline patients, echocardiographic evaluation is warranted to customize sedation protocols and fluid therapy plans to avoid adverse events including decompensated CHF and worsening outflow tract obstruction in cases of hypertrophic obstructive cardiomyopathy. Due to the concern for potential myocarditis given initial echocardiographic findings, cTnI was recommended in the Tubbs population to guide therapy. Furthermore, given the high incidence of SEC ${\rm{\pm }}$ T on echocardiographic evaluation, coagulation assessment was warranted to guide therapeutic intervention for prevention of potentially detrimental thromboembolic complications with medications such as clopidogrel or aspirin.

### Control cats

Thirty-five clinically healthy cats, that were not exposed to wildfires, were prospectively recruited from referring veterinarians, veterinary students, and hospital staff from 2017–2019. Cats receiving medications considered to have cardiovascular effects were excluded. Complete echocardiograms were performed at one timepoint in these cats. Echocardiograms were measured by a single observer (ANS).

### Echocardiography

All echocardiograms were performed by a board-certified cardiologist (JAS, CGH) or a cardiology resident-in-training (ANS) under the direct supervision of a board-certified cardiologist using a 12–4 mHz sector array transducer (Philips iE33 Ultrasound, Philips Healthcare, Andover, MA). Each cat was gently restrained in right lateral and then left lateral recumbency; no sedatives were employed. Two-dimensional (2D), M-mode, color Doppler, and spectral Doppler echocardiographic images were obtained in standard imaging planes^54^. All measurements were then performed by 1 of 3 investigators (ANS, CGH, JAS) using a commercially available offline workstation (Syngo Dynamic Workplace, Version 10.0.01_HF04_Rev5 [Build 2884], Siemens Medical Solutions, Malvern Pennsylvania). Two-dimensional right parasternal long axis imaging plane was used to obtain the maximum 2D thickness of the interventricular septum (IVSd) and left ventricular posterior wall (LVPWd) in diastole using an inner-edge to inner-edge measuring technique. M-mode was used to measure the IVSd and LVPWd as well as the LVIDd and the left ventricular internal dimension at end-systole (LVIDs). Fractional shortening (FS%) was calculated using the equation (LVIDd-LVIDs)/LVIDd x 100. The maximal LV wall thickness used for analysis was considered the greatest measurement of LV thickness in diastole among all LV measurement methods. A diastolic wall thickness $\ge$ 6.0 mm was considered consistent with MT. Diastolic wall thickness measuring 5.5 mm to 5.9 mm was considered equivocal for MT. Patients with LV thickness $\ge$ 5.5 mm and LVIDd $\le$ 1.22 cm were considered to have possible pseudohypertrophy from hypovolemia and were excluded from the MT group. A FS% < 28%^55^ was considered consistent with systolic dysfunction. Relative systolic dysfunction was defined as a normal FS% (28–63%)^55,56^ with concurrent increase in LVIDd (>1.62 cm)^56^. This finding was also considered consistent with a DCM phenotype.

Left atrial size was measured in 2D on the right parasternal short-axis basilar view to determine the LA/Ao as previously described^57^. Left atrial enlargement was defined as a long axis measurement $\ge$ 1.6 cm or LA/Ao in short axis ≥1.6 cm. The left auricular flow velocity was obtained in an oblique left apical parasternal long axis view with the pulsed wave Doppler sample volume positioned at the entrance to the left auricle. A left auricular flow velocity ≤25  m/sec was considered a risk factor for development of SEC $\pm$ T^58^. Heart rate was calculated with using an instantaneous measurement of the R-to-R interval on M-mode images.

Subjective assessment and descriptions were made by each investigator for additional findings including: SEC $\pm$ T, valvular regurgitation as determined by color Doppler interrogation, and hyperechogenicity of papillary muscles or endomyocardium. Spontaneous echocardiographic contrast was defined as a dynamic, organized swirling pattern visualized within the cardiac chambers in the absence of exogenous contrast media^48^. The cardiac chamber containing SEC, as well as the presence or absence of intracardiac thrombi were noted. A diagnosis of CHF was made on the basis of clinical evaluation in addition to echocardiographic findings of left atrial enlargement or biatrial enlargement and presence of pericardial effusion and/or pleural effusion. These findings were corroborated with evidence of pulmonary edema on thoracic radiographs in some cases.

### Postmortem examination

For cats that died or were euthanized, a necropsy was performed if owners could be contacted and consented (n = 5). Cause of death was determined and considered to be cardiac if clinical evidence of severe systolic dysfunction or CHF (atrial enlargement with cavitary effusion or pulmonary edema) was present prior to death or necropsy findings consistent with myocarditis or myonecrosis were noted. Arrhythmias and sudden death with no systemic explanation were also included as cardiac causes of death. All other causes of death were considered to be non-cardiac in origin.

### Cardiac troponin I

Cardiac troponin I was performed in the Tubbs fire cohort to evaluate for evidence of post-burn cardiac injury. Approximately 1–3 ml of whole blood were collected from a peripheral vein and placed in vacutainer tubes without anticoagulant. The samples were allowed to clot at room temperature. Serum was obtained after centrifugation and supernatant extraction. Samples were run within 1 hour on a previously validated bedside point-of-care system with an analytic sensitivity of 0.02 ng/mL^59^. A cTnI greater than 0.16 ng/ml was considered elevated^32^.

### Statistical analysis

Statistical analysis was performed using commercially available software (GraphPad Prism, MedCalc, Microsoft Excel). Data was visually inspected and tested for normality using D’Agostino Pearson Omnibus normality test. Data from wildfire exposed cats was then compared to control cats using an unpaired t-test for normally distributed data or a Mann-Whitney test for nonparametric data. For the comparison of MT between groups, the maximum wall thickness from the first evaluation was used for the exposed group. Continuous data is presented as a mean $\pm$ standard deviation and non-parametric data is presented as a median with IQR (25^th^ and 75^th^ percentiles). Categorical data were coded as 0 (no) and 1 (yes) for statistical analysis. This data was compared by constructing a 2 × 2 contingency table and analyzed by Fisher’s Exact test. Statistical significance was defined as P < 0.05.

Relative risk for development of MT or SEC $\pm$ T was assessed using MedCalc commercially available software to compare the clinically healthy population to the population exposed to the wildfires. Relative risk values are accompanied by their 95% confidence interval (CI). Statistical significance was defined as P < 0.05.

## Supplementary information

Supplementary information.

## References

[1] Vaughn L, Beckel N (2012). Severe burn injury, burn shock, and smoke inhalation injury in small animals. Part 1: Burn classification and pathophysiology. J. Vet. Emerg. Crit. Care.

[2] Vaughn L, Beckel N, Walters P (2012). Severe burn injury, burn shock, and smoke inhalation injury in small animals. Part 2: diagnosis, therapy, complications, and prognosis..

[3] Pavletic MM, Trout NJ (2006). Bullet, Bite, and Burn Wounds in Dogs and Cats. Vet. Clin. North Am. - Small Anim. Pract..

[4] Mullally C, Carey K, Seshadri R (2010). Use of a nanocrystalline silver dressing and vacuum-assisted closure in a severely burned dog. J. Vet. Emerg. Crit. Care.

[5] Quist, E. M., Tanabe, M., Mansell, J. E. & Edwards, J. L. A case series of thermal scald injuries in dogs exposed to hot water from garden hoses (garden hose scalding syndrome). *Vet. Dermatol*. **23** (2012). 10.1111/j.1365-3164.2011.01015.x 22132799

[6] Silverstein, D. C. & Hopper, K. Thermal Burn Injury. In *Critical Care Medicine* 743–747 (Elsevier, 2009).

[7] Stawski C, Doty AC (2019). A physiological understanding of organismal responses to fire. Curr. Biol..

[8] California Department of Forestry and Fire Protection. Top 20 Deadliest California Wildfires. 1. Available at: https://www.fire.ca.gov/media/5512/top20_deadliest.pdf (2019).

[9] Williams FN (2011). Changes in cardiac physiology after severe burn injury. J. Burn Care Res..

[10] Adams GA (2004). Macrophage migration inhibitory factor mediates late cardiac dysfunction after burn injury. Am. J. Physiol. Circ. Physiol..

[11] Suzuki K, Nishina M, Ogino R, Kohama A (2017). Left ventricular contractility and diastolic properties in anesthetized dogs after severe burns. Am. J. Physiol. Circ. Physiol..

[12] Clementi Emily, Talusan Angela, Vaidyanathan Sandhya, Veerappan Arul, Mikhail Mena, Ostrofsky Dean, Crowley George, Kim James, Kwon Sophia, Nolan Anna (2019). Metabolic Syndrome and Air Pollution: A Narrative Review of Their Cardiopulmonary Effects. Toxics.

[13] Guillory, A. N., Clayton, R. P., Herndon, D. N. & Finnerty, C. C. Cardiovascular dysfunction following burn injury: What we have learned from rat and mouse models. *Int. J. Mol. Sci*. **17** (2016). 10.3390/ijms17010053 PMC4730298 26729111

[14] Abu-Sittah GS, Sarhane KA, Dibo SA, Ibrahim A (2012). Cardiovascular dysfunction in burns: Review of the literature. Ann. Burns Fire Disasters.

[15] Jeschke Marc G., Gauglitz Gerd G., Kulp Gabriela A., Finnerty Celeste C., Williams Felicia N., Kraft Robert, Suman Oscar E., Mlcak Ronald P., Herndon David N. (2011). Long-Term Persistance of the Pathophysiologic Response to Severe Burn Injury. PLoS ONE.

[16] Zhang JP (2008). Apoptosis in cardiac myocytes during the early stage after severe burn. J. Trauma - Inj. Infect. Crit. Care.

[17] Kenyon NJ (2017). Early Life Wildfire Smoke Exposure Is Associated with Immune Dysregulation and Lung Function Decrements in Adolescence. Am. J. Respir. Cell Mol. Biol..

[18] Walker PF (2015). Diagnosis and management of inhalation injury: An updated review. Crit. Care.

[19] Wohlsein P, Peters M, Schulze C, Baumgärtner W (2016). Thermal injuries in eterinary Forensic pathology. Vet. Pathol..

[20] Lavrentieva A, Depetris N, Kaimakamis E, Berardino M, Stella M (2016). Monitoring and treatment of coagulation abnormalities in burn patients. An international survey on current practices. Ann. Burns Fire Disasters.

[21] Barret JP, Dziewulski PG (2006). Complications of the hypercoagulable status in burn injury..

[22] Prat Nicolas J., Herzig Maryanne C., Kreyer Stefan, Montgomery Robbie K., Parida Bijaya K., Linden Katharina, Scaravilli Vittorio, Belenkiy Slava M., Cancio Leopoldo C., Batchinsky Andriy I., Cap Andrew P. (2017). Platelet and coagulation function before and after burn and smoke inhalation injury in sheep. Journal of Trauma and Acute Care Surgery.

[23] Sithu SD (2010). Exposure to acrolein by inhalation causes platelet activation. Toxicol. Appl. Pharmacol..

[24] Younan D (2017). Echocardiographic correlates are associated with in-hospital mortality in trauma and burn patients. Am. J. Surg..

[25] Maybauer MO (2014). Transesophageal echocardiography in the management of burn patients. Burns.

[26] Bak Z, Sjöberg F, Eriksson O, Steinvall I, Janerot-Sjoberg B (2008). Cardiac dysfunction after burns. Burns.

[27] Maass DL, Naseen RH, Garry M, Horton JW (2006). Echocardiography assessment of myocardial function after burn injury. Shock.

[28] Alexander W, Schneider HG, Smith C, Cleland H (2018). The incidence and significance of raised troponin levels in acute burns. J. Burn Care Res..

[29] Zeng L, Chen Y, Wu M (2001). Cardiac troponin I: a marker for detecting non-ischemic cardiac injury. Zhonghua Yi Xue Za Zhi.

[30] Langhorn R, Willesen JL (2016). Cardiac troponins in dogs and cats. J. Vet. Intern. Med..

[31] Chen YN (2000). Cardiac troponin I: a marker for post-burn cardiac injury. Ann. Clin. Biochem..

[32] Sleeper M, Clifford C, Laster L (2001). Cardiac troponin in the normal dog and cat. J. Vet. Intern. Med..

[33] Ettinger, S. J. & Feldman, E. C. Textbook of Veterinary Internal Medicine (2010).

[34] Maxie, G. *Jubb, Kennedy & Palmer’s Pathology of Domestic Animals*. (Saunders Ltd, 2007).

[35] Carlson Deborah L., Horton Jureta W. (2006). Cardiac Molecular Signaling After Burn Trauma. Journal of Burn Care & Research.

[36] Horton JW, Garcia NM, White DJ, Keffer J (1995). Postburn cardiac contractile function and biochemical markers of postburn cardiac injury. J. Am. Coll Surg..

[37] Paige CF, Abbott JA, Elvinger F, Pyle RL (2009). Prevalence of cardiomyopathy in apparently healthy cats. J. Am. Vet. Med. Assoc..

[38] Payne JR, Brodbelt DC, Luis Fuentes V (2015). Cardiomyopathy prevalence in 780 apparently healthy cats in rehoming centres (the CatScan study). J. Vet. Cardiol..

[39] Luis Fuentes V, Wilkie LJ (2017). Asymptomatic Hypertrophic Cardiomyopathy. Vet. Clin. North Am. Small Anim. Pract..

[40] Novo Matos J (2018). Transient Myocardial Thickening in Cats Associated with Heart Failure. J. Vet. Intern. Med..

[41] Izgi C (2015). Myocardial edema in Takotsubo syndrome mimicking apical hypertrophic cardiomyopathy: An insight into diagnosis by cardiovascular magnetic resonance. Hear. Lung J. Acute Crit. Care.

[42] Hiramitsu S (2002). Transient Ventricular Wall Thickening in Acute Myocarditis. Jpn. Circ. J..

[43] Fagin A, Sen S, Palmieri T, Greenhalgh D (2012). Takotsubo cardiomyopathy caused by severe burn injury. J. Burn Care Res..

[44] Wikiel K, Gemma LW, Yowler CJ, Coffee T, Brandt CP (2011). Takotsubo cardiomyopathy after minor burn injury. J. Burn Care Res..

[45] Kato T (2013). Two cases of reversible left ventricular hypertrophy during recovery from takotsubo cardiomyopathy. Echocardiography.

[46] Hwang HJ (2014). Evolutionary change mimicking apical hypertrophic cardiomyopathy in a patient with takotsubo cardiomyopathy. Echocardiography.

[47] O’Halloran, E. *et al*. The impact of non-severe burn injury on cardiac function and long- term cardiovascular pathology. *Nat. Publ. Gr*. 1–11. 10.1038/srep34650 (2016). 10.1038/srep34650 PMC5046146 27694999

[48] BLACK IAN W. (2000). Spontaneous Echo Contrast: Where There's Smoke There's Fire. Echocardiography.

[49] Peck, C. M., Nielsen, L. K., Quinn, R. L., Laste, N. J. & Price, L. L. Retrospective evaluation of the incidence and prognostic significance of spontaneous echocardiographic contrast in relation to cardiac disease and congestive heart failure in cats: 725 cases. *J. Vet. Emerg. Crit. Care. (San Antonio)* (2006–2011). **26**, 704–712 (2016). 10.1111/vec.12509 27479924

[50] Zotz, R. J., Müller, M., Genth-Zotz, S. & Darius, H. Spontaneous echo contrast caused by platelet and leukocyte aggregates? *Stroke* 1127–1133 (2001). 10.1161/01.str.32.5.1127 11340221

[51] Pombo M, Lamé MW, Walker NJ, Huynh DH, Tablin F (2015). TCDD and omeprazole prime platelets through the aryl hydrocarbon receptor (AhR) non-genomic pathway. Toxicol. Lett..

[52] Chetboul V., Concordet D., Pouchelon J. L., Athanassiadis N., Muller C., Benigni L., Munari A. C., Lefebvre H. P. (2003). Effects of Inter- and Intra-Observer Variability on Echocardiographic Measurements in Awake Cats. Journal of Veterinary Medicine Series A.

[53] van Hoek I, Payne JR, Feugier A, Connolly DJ (2018). Inter-observer variability for cardiac ultrasound measurements in cats repeated at different time points in early adult life. Vet. Anim. Sci..

[54] Boon, J. Myocardial Diseases. In *Veterinary Echocardiography* 359–410 (Wiley-Blackwell, 2011).

[55] Häggström J (2016). Effect of Body Weight on Echocardiographic Measurements in 19,866 Pure-Bred Cats with or without Heart Disease. J. Vet. Intern. Med..

[56] Schober K, Savino S, Yildiz V (2017). Reference intervals and allometric scaling of two-dimensional echocardiographic measurements in 150 healthy cats. J. Vet. Med. Sci..

[57] Abbott, J. A. & MacLean, H. N. Two-Dimensional Echocardiographic assessment of the feline left atrium. *J. Vet. Intern. Med*. 111–119 (2006). 10.1892/0891-6640(2006)20[111:teaotf]2.0.co;2 16496930

[58] Schober KE, Maerz I (2006). Assessment of left atrial appendage flow velocity and its relation to spontaneous echocardiographic contrast in 89 cats with myocardial disease. J. Vet. Intern. Med..

[59] Adin Darcy B., Milner Rowan J., Berger Kate D., Engel Cathy, Salute Marc (2005). Cardiac troponin I concentrations in normal dogs and cats using a bedside analyzer. Journal of Veterinary Cardiology.

